# Evaluation of the Patient Acceptable Symptom State (PASS) in Italian Patients Affected by Systemic Lupus Erythematosus: Association with Disease Activity Indices

**DOI:** 10.1371/journal.pone.0073517

**Published:** 2013-09-09

**Authors:** Fabrizio Conti, Fulvia Ceccarelli, Laura Massaro, Viviana A. Pacucci, Francesca Miranda, Simona Truglia, Enrica Cipriano, Francesco Martinelli, Ilaria Leccese, Francesca Romana Spinelli, Cristiano Alessandri, Carlo Perricone, Guido Valesini

**Affiliations:** Lupus Clinic, Reumatologia, Dipartimento di Medicina Interna e Specialità Mediche, Sapienza Università di, Roma, Rome, Italy; University of Michigan Medical School, United States of America

## Abstract

**Objectives:**

The aim of this study was to evaluate the discriminant capability of the patient acceptable symptom state (PASS) according to disease activity, in a cohort of Italian patients affected by systemic Lupus erythematosus (SLE).

**Methods:**

Consecutive SLE patients were enrolled. At each visit, the patients underwent a complete physical examination and the clinical/laboratory data were collected in a standardized, computerized, and electronically-filled form. The evaluation of serum complement C3 and C4 levels and determination of autoantibodies was obtained. Disease activity was assessed with the SLEDAI-2K and ECLAM, while chronic damage was measured with the SLICC. Finally, PASS was assessed in all patients by asking to answer yes or no to a single question.

**Results:**

One hundred sixty-five patients were enrolled (M/F 12/153; mean age 40.4±11.8 years, mean disease duration 109.1±96.2 months). No patients refused to answer, suggesting the acceptability of PASS. A total of 80% of patients rated their state as acceptable. The patients with an acceptable status had significantly lower mean SLEDAI-2K and ECLAM scores than the others [1.8±2.7 versus 3.4±2.3(P=0.004); 0.7±0.9 versus 1.4±1.1(P=0.0027)]. No significant differences were observed when considering chronic damage, evaluated with SLICC.

**Conclusions:**

In the clinical practice, SLE patients assessment performed by using complex disease activity indices such as SLEDAI-2K and ECLAM, could be time consuming. In our study, for the first time, we used PASS, a quick and easily comprehensible tool, to evaluate the patients’ status, this single question seems to be able to discriminate patients with different disease activity, especially when this is determined by musculoskeletal involvement.

## Introduction

Systemic lupus erythematosus (SLE) is a multifactorial autoimmune disease, involving genetic and environmental factors, characterized by a wide range of autoantibodies and clinical manifestations [1–10]. Monitoring of disease activity is an important aspect in the management of SLE patients, as recently pointed out in a core-set of recommendations proposed by the European League Against Rheumatism (EULAR) [11].

Through the years, many indices have been developed and validated to measure disease activity in SLE patients, such as the Systemic Lupus Erythematosus Disease Activity Index 2000 (SLEDAI-2K) and the European Consensus Lupus Activity Measurement (ECLAM) [12,13]. Flare is another outcome measure that identifies patients with a worsening of disease activity. Several definitions have been proposed according to the disease activity index adopted, but no consensus has been reached so far [14,15]. More recently, in order to identify patients with a disease course characterized by a persistent status of activity, the concept of persistently active disease (PAD) was proposed [16–18].

Furthermore, in daily clinical practice, evaluation of disease activity is not always feasible due to time consuming and lacking data. Thus, it could be of interest the development of a feasible and time-sparing tool to assess patients’ status.

As recently pointed-out, the Outcome Measures in Rheumatology Clinical Trials (OMERACT) recommended the measurement of patient well-being, identified by a dichotomous conditions: satisfactory versus unsatisfactory status [19,20].

The patient acceptable symptom state (PASS) is a single-question outcome tool to evaluate the level of symptoms at which patients consider themselves well [21]. Data published in the literature report the application of PASS to patients affected by Ankylosing Spondylitis (AS), osteoarthritis (OA), and Rheumatoid Arthritis (RA). All these studies have demonstrated a significant association between PASS and disease activity, evaluated with different indices [22–26].

However, no data are available concerning a possible application of PASS in patients affected by SLE. Thus, the aim of the present study was to evaluate the discriminant capability of PASS according with disease activity in a cohort of Italian SLE patients.

## Materials and Methods

Consecutive SLE patients were enrolled between January 2010 and June 2012 at the Lupus Clinic of the Rheumatology Unit, Sapienza University of Rome (“Sapienza Lupus Cohort”).

SLE diagnosis was performed according to the revised 1997 American College of Rheumatology (ACR) criteria [27]. Patients provided written informed consent at the time of the visit. The local ethical committee of “Policlinico Umberto I” of Rome, approved the study.

At each visit, the patients underwent a complete physical examination, the clinical and laboratory data were collected in a standardized, computerized, and electronically-filled form, which includes demographics, education level, past medical history with date of diagnosis, co-morbidities, previous and concomitant treatments.

The evaluation of serum complement C3 and C4 levels and determination of autoantibodies was obtained. ANA were determined by means of indirect immunofluorescence (IIF) on HEp-2 (titer ≥1:160 or ++ on a scale from + to ++++); anti-dsDNA with ELISA assays (considering levels two folds higher than the cut-off of the reference laboratory) or IIF on *Crithidia* Luciliae (titer ≥1:10), ENA (including anti-Ro/SSA, anti-La/SSB, anti-Sm, anti-RNP) by ELISA assay considering titers above the cut-off of the reference laboratory, anti-CL of IgG or IgM isotype, by ELISA, in serum or plasma at medium or high titers (e.g., > 40 GPL or MPL or above the 99^th^ percentile), anti-β_2_Glycoprotein-I (GPI) of IgG or IgM isotype, by ELISA, in serum or plasma (above the 99^th^ percentile), and finally Lupus anticoagulant (LA) was assessed according to the guidelines of the International Society on Thrombosis and Hemostasis (Scientific Subcommittee on lupus anticoagulant/phospholipid-dependent antibodies) [28]. For all the subjects, C3 and C4 concentrations were studied by means of radial immunodiffusion. Urine protein analysis was performed in patients with renal involvement (proteinuria/24 hours).

Disease activity was assessed at each visit with the SLEDAI-2K and ECLAM, while chronic damage was measured by the System Lupus International Collaborating Clinics (SLICC) Damage Index (SDI) [12,13,29].

In patients with more than 2 consecutive visits with a minimum interval of 2 months between them, the assessment of PAD could be performed and PAD was defined as a SLEDAI-2K score > 4, excluding serology alone [16].

In a subgroup of the enrolled patients the evaluation of quality of life by using the Medical Outcomes Study-Short Form 36 (SF-36) was performed [30].

### Patient acceptable symptom state evaluation

PASS was assessed in all patients by asking to answer yes or no to a single question [21] that was previously translated into Italian. In the linguistic validation process, two Italian translators, who are native speakers and are experienced in translating health questionnaires, independently translated the question. After this process, the two translators compared their translations and produced a third translation jointly, which was given to a native English-speaking translator for translation back into English. Finally, the question was compared with the original English version and approved.

The original question was "Considering all the different ways your disease is affecting you, if you would stay in this state for the next months, do you consider that your current state is satisfactory?” [21]. For this question patients could give a dichotomized answer (yes or no). The Italian version administered to our SLE cohort was reported in Table S1.

The discriminant ability of PASS for use in SLE patients was assessed by comparing the relationship of PASS responses (yes *versus* no) with the disease activity indices SLEDAI-2K, ECLAM and PAD, recorded during the same visit. Moreover, the association with the chronic damage, assessed by SDI, was evaluated.

### Statistical evaluation

We used version 13.0 of the SPSS statistical package. Normally distributed variables were summarized using the mean ± SD, and non-normally distributed variables by the median and range. Mann-Whitney test was performed accordingly. Univariate comparisons between nominal variables were calculated using chi-square test or Fisher-test where appropriate. All the associations were referred to the last visit at which PASS was evaluated. Two-tailed P values were reported, P values less than 0.05 were considered significant.

## Results

One hundred sixty-five patients (M/F 12/153; mean age 40.4±11.8 years, mean disease duration 109.1±96.2 months; mean SLEDAI-2K 2.1±2.8; mean ECLAM 0.8±1.0; mean SDI 0.2±0.6) were evaluated.

No patients refused to answer, suggesting the acceptability of PASS. In our cohort, 136 patients (82.4%) showed a high educational level (more than 8 years of education), while 29 patients showed a low level of education (less than 8 years of education). No patients asked the examiner to repeat the question due to non-comprehension, and there was no difference in the percentage of patients answering yes or no according to the educational level (high level of education: yes 78.7%, no 21.3%; low level of education: yes 86.2%, no 13.8%, P=0.26), suggesting that the question was easy understood.

According with the answer given to PASS, the patients were dichotomized in group 1 (patients answering yes) and group 2 (patients answering no).

In table 1, the main demographic, clinical and laboratory features of SLE patients are reported according with the answer to PASS.

**Table 1:** Main demographic, clinical and laboratory features of 165 SLE patients, sub-grouped according with the response to PASS question.

	***PASS-Yes***	***PASS-No***	
	***(Group1N=132)***	***(Group2N=33)***	***Pvalue***
**Mean age±SD (years)**	40.2+11.5	41.2+13	0.09
**Sex (M/F)**	11/121	1/32	0.08
**Mean disease duration ± SD (months)**	96+98.8	81.9+80.4	0.07
**Clinical manifestations (N/%)**			
Mucocutaneous manifestations (*malar/discoid rash, photosensitivity, oral ulcers*)	12/9.2	4/12.1	0.05
Arthritis	10/7.7	13/39.3	0.00003
Serositits (*Pleuritis or pericarditis*)	0	1/3.0	0.16
Renal involvement (*proteinuria >0.5gr/day or cellular casts*)	7/5.3	2/6	1
Hematological manifestations (*hemolytic anemia, leukopenia, lymphopenia, thrombocytopenia*)	17/13	8/24	0.1
Neurological involvement (*seizures or psychosis*)	3/2.2	1/3	0.1
**Laboratory assessment (N/%)**			
ANA	93/89	24/92	0.8
Anti-dsDNA	36/38.7	8/32	0.8
anti-Sm	2/2.3	3/21	0.05
anti-RNP	2/2.3	2/14	0.002
anti-SSA/Ro	16/18.2	6/42	0.003
anti-SSB/La	5/5.6	1/7.1	1
anti-CL	19/29	4/28	1
Anti-β2GPI	13/27	1/10	0.003
LA	11/23	1/10	0.02
Mean C3 levels ± SD (mg/dl)	95.6+26.9	87.4+23.8	0.1
Mean C4 levels ± SD (mg/dl)	18.1+11.2	14.9+6.8	0.2
**Therapy**			
Glucocorticoids (N/%)	96/72.7	26/78.7	0.6
Mean glucocorticoids dosage±SD (prednisone equivalents) (mg/weekly)	48.2+38.5	51.8+36.1	0.4
Hydroxychloroquine (N/%)	93/70	21/63	0.5
**Immunosuppressant agents (N/%)**	43/32.5	16/48.4	0.03
Methotrexate (N/%)	10/7.6	3/9.1	0.7
Cyclosporine A (N/%)	13/9.8	7/21.2	0.1
Mycophenolate Mofetil (N/%)	24/18.2	6/18.2	1
Cyclophosphamide (N/%)	1/0.7	0	1
Azathioprine (N/%)	34/25.7	8/24.2	1
Rituximab (N/%)	1/0.7	0	1

Abbreviations. SD: Standard deviation; CL: cardiolipin; LA: Lupus Anticoagulant.

**Figure 1:**
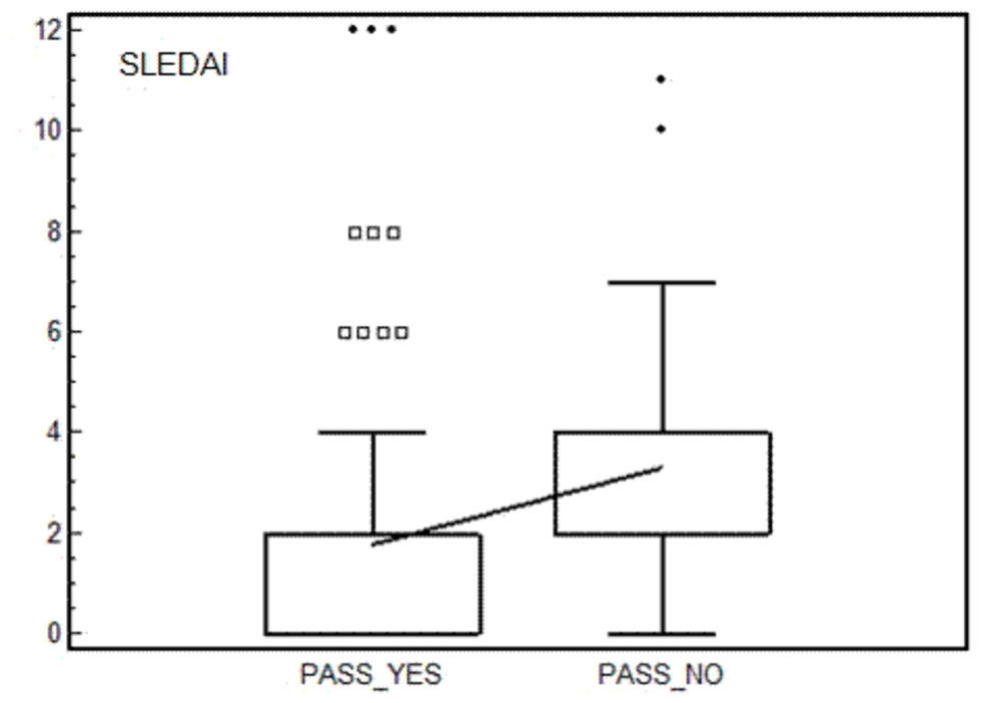
SLEDAI values according with answer given to PASS question. Box and whiskers plot (median, quartiles, range and possible extreme values).

**Figure 2:**
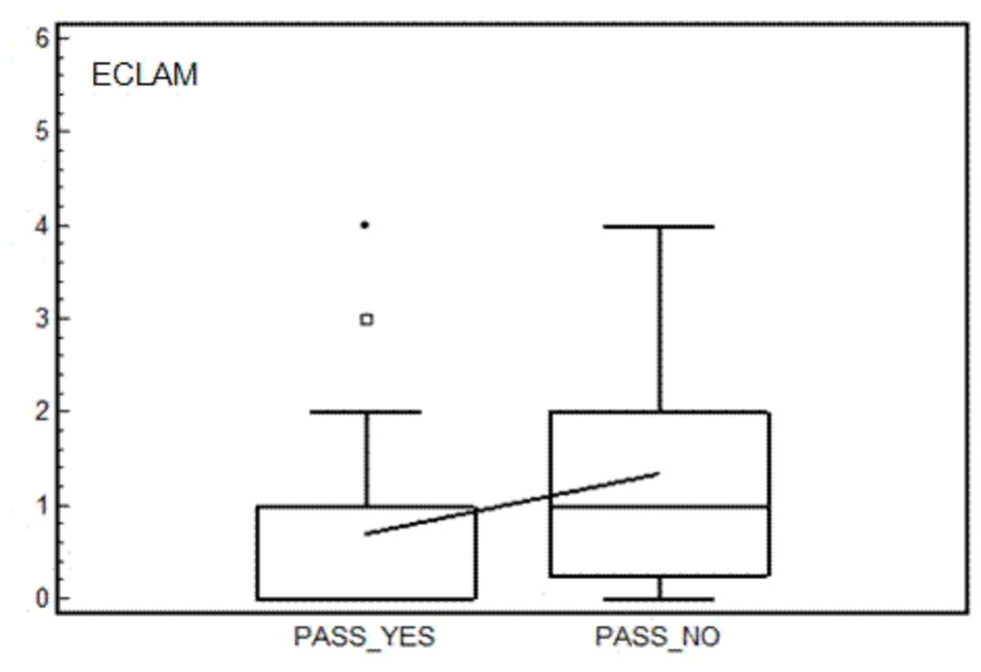
ECLAM values according with answer given to PASS question. Box and whiskers plot (median, quartiles, range and possible extreme values).

Interestingly, group 1 patients had significantly lower mean SLEDAI-2K and ECLAM scores than group 2 patients [1.8±2.7 versus 3.4±2.3(P=0.004); 0.7±0.9 versus 1.4±1.1(P=0.0027), respectively; Figure 1 and Figure 2]. Although not statistically significant, the percentage of patients with PAD was lower in group 1 than group 2 patients (14.4% *versus* 21.1%, P=0.2).

No significant differences were observed when considering chronic damage, evaluated with SDI (group 1: 0.2±0.6, group 2: 0.2±0.5; P=0.09).

Aiming at evaluating the impact of the quality of life on PASS status, 30 patients (M/F 2/28; mean age ± SD 39.8±11.8 years; mean disease duration ± SD 112.4±102.7 months) completed the SF-36 questionnaire. Among these, 21 patients (70%) answered yes to PASS question. In table 2 we reported the mean values of SF-36 items according to PASS status. Patients answering yes showed higher values of all items, except for mental health. A significant difference between the two groups was demonstrated when considering the items “vitality” and “role limitations due to emotional problems” (P=0.01 and P=0.03, respectively).

**Table 2:** SF-36 items according with answer to PASS question.

***SF-36Domain*** (mean±SD)	***PASS-Yes***	***PASS-No***	***P***
	***(N=21)***	***(N=9)***	
Physical functioning	78.7±24.7	63.8±26.3	0.05
Role limitations due to physical problems	66.4±43.4	51.2±43.4	0.09
Bodily Pain	69.4±27.4	47.3±21.6	0.08
General health perceptions	52.0±24.7	67.4±17.1	0.07
Vitality	62.7±25.3	42.2±16.2	0.01
Social Functioning	72.1±24.9	62.2±19.7	0.1
Role limitations due to emotional problems	64.6±43.3	36.2±41.8	0.03
Mental health	70.0±21.4	89.8±27.7	0.09

Abbreviations. SF-36: Short-Form 36; SD: Standard deviation;

## Discussion

In the present study, we demonstrated for the first time the ability of the Patient Acceptable Symptom State (PASS) in discriminating patients affected by SLE with different level of the disease activity.

As emphasized during the OMERACT 6 meeting, the patients’ perspective evaluation is a very important outcome to perform a complete assessment, and it could influence clinical decision making [19]. In this context, PASS is a simple, reliable, and valid assessment of well-being and could be easily applied in rheumatology practice. This is a single and quick question, requiring little amount of time to be answered, due to the presence of a Boolean response (yes or no).

So far, PASS was applied in patients affected by different rheumatic conditions, such as OA, AS, and RA, showing a significant correlation with disease activity [22–26]. At first, PASS was evaluated in patients with knee and hip OA after a 4-weeks period of treatment with NSAIDs. PASS-defined satisfactory status was recorded in 57.7% of patients with knee OA and 50.2% with hip OA [23]. The percentage of patients with knee OA giving a positive response to PASS was lower in a study by Dougados and coll. characterized by a longer follow-up (13 weeks follow-up) in which the response to different Coxib was evaluated [31].

In patients affected by AS, a significant association between the presence of an acceptable symptom state and a reduced disease activity, assessed with BASDAI, BASFI and/or ASDAS, was found, underlining the external validity of PASS [32–34]. Rodriguez-Lozano et al. evaluated the discriminant capacity of PASS showing that AS patients with positive response to PASS assumed significantly lower dosages of NSAIDs and steroids than those patients not achieving an acceptable symptom state [23]. Similarly, a significantly higher frequency of patients with an acceptable status was achieved by AS patients treated with adalimumab compared with patients assuming placebo. Interestingly, patients treated with adalimumab had also lower disease activity [21,22,35].

More recently, PASS was administered to patients affected by RA. It has been shown that a positive response to PASS is associated with a range of moderate disease activity assessed with several composite indices, such as DAS28, CDAI and SDAI [36,37].

Following these previous experiences, we evaluated the discriminant capability of PASS in a cohort of Italian SLE patients. A significant association with disease activity, assessed by SLEDAI-2K and ECLAM was identified. In particular, patients with a positive response to PASS showed significant lower levels of SLEDAI-2K and ECLAM. In this same group, a lower percentage of patients with persistently active disease, defined by PAD, was observed.

When analyzing the clinical manifestations, a significant lower frequency of musculoskeletal involvement was found in patients with an acceptable symptom state. Joint involvement in SLE is very common, affecting up to 90% of patients at any stage of disease. The clinical presentation of joint involvement can widely vary, ranging from arthralgia, without erosions or deformity, to an erosive arthritis with severe functional disability [38]. The results of the present study confirm the impact of the musculoskeletal involvement in the acceptability of the patient’s status. The disability linked to the joint involvement, associated with the inability to perform the daily activities and the possible need of caregivers help, could make unacceptable the status of a SLE patient.

In our cohort, a significant higher percentage of autoantibodies in patients with a negative response to PASS was found. We could hypothesize that the presence of autoantibodies might be associated with a greater disease activity, influencing the acceptability of the patient’s status. Indeed, it’s widely known the association between anti-SSA and anti-RNP antibodies and mucocutaneous involvement in SLE patients [39]. Since body image is a major theme identified by patients, it is not surprising that mucocutaneous involvement and in turn the presence of anti-SSA and anti-RNP antibodies were more frequent in patients not accepting their status [40].

The absence of an association between PASS and SDI in this cohort is not surprising. Indeed, the response to PASS freezes the actual disease status, reflected by the disease activity but not by chronic damage indices. Moreover, in our population the low mean values of SDI probably don’t influence the patient’s status.

Even though SF-36 was administered only to a subgroup of SLE patients, overall a worse quality of life was identified in the patients answering no to PASS question.

## Conclusions

PASS is a simple, reliable, and valid patient-reported outcome to assess patients’ well-being. It could provide a highly feasible tool for clinicians to focalize the disease activity status in SLE patients. In daily clinical practice PASS should not be applied as replacement but administered together with the commonly used activity indices. Larger studies are needed to confirm these results and verify its actual application in a disease characterized by a great clinical heterogeneity, such as SLE.

## Supporting Information

Table S1 **Translation to Italian language of original question of patient acceptable symptom state (PASS).**
(DOC)
