# Supplementary material for: Evaluation of the Patient Acceptable Symptom State (PASS) in Italian Patients Affected by Systemic Lupus Erythematosus: Association with Disease Activity Indices
Source: PLoS One. 2013 Sep 9;8(9):e73517. doi: 10.1371/journal.pone.0073517 (PMC3767687; doi:10.1371/journal.pone.0073517)
Supplement: Table S1 — Translation to Italian language of original question of patient acceptable symptom state (PASS). (DOC) [file pone.0073517.s001.doc]

| ***Original question*** | Considering all the different ways your disease is affecting you, if you would stay in this state for the next months, do you consider that your current state is satisfactory? |
| --- | --- |
| ***Italian version*** | Considerando complessivamente lo stato attuale della sua malattia, se dovesse rimanere stabile nei prossimi mesi, riterrebbe la sua condizione soddisfacente? |

**Table S1:** Translation to Italian language of original question of patient acceptable symptom state (PASS)
